# Development of CS-TPP-dsRNA nanoparticles to enhance RNAi efficiency in the yellow fever mosquito, *Aedes aegypti*

**DOI:** 10.1038/s41598-019-45019-z

**Published:** 2019-06-19

**Authors:** Ramesh Kumar Dhandapani, Dhandapani Gurusamy, Jeffrey L. Howell, Subba Reddy Palli

**Affiliations:** Department of Entomology, University of Kentucky, Lexington, Kentucky 40546 USA

**Keywords:** Entomology, RNAi

## Abstract

Mosquito-borne diseases are a major threat to human health and are responsible for millions of deaths globally each year. Vector control is one of the most important approaches used in reducing the incidence of these diseases. However, increasing mosquito resistance to chemical insecticides presents challenges to this approach. Therefore, new strategies are necessary to develop the next generation vector control methods. Because of the target specificity of dsRNA, RNAi-based control measures are an attractive alternative to current insecticides used to control disease vectors. In this study, Chitosan (CS) was cross-linked to sodium tripolyphosphate (TPP) to produce nano-sized polyelectrolyte complexes with dsRNA. CS-TPP-dsRNA nanoparticles were prepared by ionic gelation method. The encapsulation efficiency, protection of dsRNA from nucleases, cellular uptake, *in vivo* biodistribution, larval mortality and gene knockdown efficiency of CS-TPP-dsRNA nanoparticles were determined. The results showed that at a 5:1 weight ratio of CS-TPP to dsRNA, nanoparticles of less than 200 nm mean diameter and a positive surface charge were formed. Confocal microscopy revealed the distribution of the fed CS-TPP-dsRNA nanoparticles in midgut, fat body and epidermis of yellow fever mosquito, *Aedes aegypti* larvae. Bioassays showed significant mortality of larvae fed on CS-TPP-dsRNA nanoparticles. These assays also showed knockdown of a target gene in CS-TPP-dsRNA nanoparticle fed larvae. These data suggest that CS-TPP nanoparticles may be used for delivery of dsRNA to mosquito larvae.

## Introduction

Mosquitoes affect human health by transmitting deadly diseases such as malaria, Chikungunya, dengue, and yellow fever. These mosquito-borne pathogens negatively affect millions of people each year, and the burden of diseases transmitted by mosquitoes on human health has been well documented^1^. Vector control is a crucial strategy to mitigate transmission and reduce the prevalence of vector-borne diseases. Conventional methods for adult mosquito control include repellents, insecticidal sprays, and insecticide-treated nets. Management of larvae is typically limited to insecticidal treatments and removal of standing water sources in areas where mosquito populations are abundant. However, the efficacy of these methods has been decreasing over time due to the widespread development of resistance by mosquitoes to chemical insecticides and growing environmental concerns of residual toxicity^2^. In this context, RNA interference (RNAi), a sequence-specific, post-transcriptional gene silencing method that is triggered by the introduction of double-stranded RNA (dsRNA) may help^3^. RNAi technology has emerged not only as a powerful tool for functional genomics studies but also as a potentially useful method for controlling insect pests and disease vectors because of its target specificity. Although RNAi is a highly conserved mechanism in eukaryotes including fungi, plants, insects, and mammals^4–9^, there are still hurdles for successful implementation of RNAi to knockdown genes in many species of insects^10^. Lack of suitable delivery methods for dsRNA and variability in cellular uptake and transport of dsRNA in insects are the significant challenges in gene silencing efforts. Additionally, dsRNA must overcome specific obstacles such as poor cellular internalization, rapid degradation by nucleases, and limited blood stability^11^. Another limitation for successful RNAi in insects is the lack of conserved dsRNA transporter genes, resulting in a poor systemic RNAi response^12^. Also, dsRNA is prone to degradation by nucleases in the body of the insect^13^. Therefore, developing a safe and effective dsRNA carrier system for delivery into target cells of the cytoplasm is critical in order to investigate its potential application for control of insect pests and disease vectors.

Among the vectors that have been studied for delivery of genes into cells, viral vectors have demonstrated high transfection efficiency in most cells studied. However, non-viral vectors are much more attractive due to their ease of synthesis, low immune response against the vector, unrestricted load carrying capacity and safety^14^. A chitosan-based carrier system is one example of a non-viral vector that has gained interest in recent years as a safe and economical delivery system for dsRNA, siRNA, plasmid DNA (pDNA), oligonucleotides (ODN), peptides and even proteins. CS has beneficial properties such as low toxicity, low immunogenicity, and excellent biodegradability as well as a high positive charge that helps to form polyelectrolyte complexes with negatively charged nucleic acids by electrostatic interaction^15–19^. CS-based nanoparticles have been successfully used for delivery of dsRNA in mosquitoes^20–22^. However, the knockdown efficiency achieved by CS conjugated dsRNAs is not very high. Therefore, there is a need to improve CS nanoparticles for efficient delivery of dsRNA. Cross-linkers such as sodium tripolyphosphate (STPP) could play an important role in stabilizing CS nanoparticles. Sodium tripolyphosphate (STPP) is a suitable cross-linker due to its small size, anionic charge, low toxicity and quick gelling ability^23^. A CS-siRNA complex can be formulated through several cross-linkers, such as TPP, dextran sulfate, and poly-D-glutamic acid using ionic gelation methodology. Due to its small size, high rate of entrapment and binding affinity, CS-TPP nanoparticles are ideal for siRNA delivery. Indeed, serum stability assays exhibit higher siRNA protection when CS-TPP nanoparticles were used^24^. CS-TPP nanoparticles were shown as better vehicles of siRNA delivery when compared to CS-siRNA complexes, and this complex was easily taken up by cells^25^. Thus, CS-TPP-dsRNA can be used for gene delivery into cells. In this study, positively charged CS-TPP-dsRNA nanoparticles were prepared to deliver dsRNA to induce RNAi in the yellow fever mosquito, *Aedes aegypti*.

## Results and Discussion

### Synthesis of CS-TPP-dsRNA nanoparticles

The CS-TPP-dsRNA nanoparticles were prepared by ionic gelation method by cross-linking the negatively charged phosphate groups of TPP with the positively charged amino groups of CS. Visual inspection of CS-TPP-dsRNA solution showed a clear solution when TPP concentration is low (0.25-0.75 mg/ml), opalescent suspension when TPP concentration is medium to high (0.75–1.5 mg/ml) and spontaneous aggregation under high TPP (1.25–1.5 mg/ml) and low CS (1 mg/ml) concentrations (Table S1). These results suggest that the formation of suitable nanoparticles only occurs at specific concentrations of CS and TPP^26–28^.

The nanoparticles were prepared by ionic interaction using different ratios (1:1, 3:1, 5:1 and 7:1) of CS-TPP. During the synthesis of nanoparticles, the ratio of CS-TPP was adjusted to synthesize smaller size and positively charged particles to facilitate their interaction with cell membranes. The size, charge, and PDI measured by DLS are listed in Table S2. The charge of CS-TPP nanoparticles increased with an increase in CS concentration. The size of the particles increased from CS-TPP ratio of 1:1 to 7:1 except at a ratio of 5:1 where the particle size was lower than those formed by all other ratios tested. The CS-TPP ratio of 5:1 which formed smaller particles with a positive charge were selected for further development. CS-TPP-dsRNA nanoparticles were prepared by ionotropic gelation method previously described for the development of siRNA, protein, peptides, and drug molecule nanoparticles^29–33^. CS-TPP-dsRNA nanoparticles were prepared using 250 µg of dsRNA, 1 mg TPP and 1–7 mg CS and characterized by DLS. The CS-TPP-dsRNA nanoparticles formed by increasing concentration of CS (1–7 mg) showed an increase in size except at 5 mg CS where lower size particles were detected (Table S3). The charge of the nanoparticles also increased with an increase in the concentration of CS. The incorporation of dsRNA into CS-TPP-dsRNA nanoparticles increased with an increase in the concentration of CS from 1–5 mg but decreased at 7 mg CS (Table S3). The CS-TPP-dsRNA nanoparticles were analyzed by gel retardation assays. As shown in Fig. S1, the dsRNA did not form complexes with CS-TPP at 1:1 ratio of CS-TPP. In contrast, at CS-TPP ratio of 3:1, 5:1 and 7:1 nanoparticles were formed and showed a retarded migration and stayed in the well (Fig. S1). The results showed that 3:1, 5:1 and 7:1 ratio of CS-TPP induced the formation of complexes with dsRNA. This may be due to the formation of nanoparticles in a process derived from inter and intramolecular linkages facilitated by the anionic molecules^34^. The nanoparticles produced using dsRNA and CS:TPP at 3:1, 5:1 and 7:1 ratio were tested in Aag-2 mosquito cell line. The nanoparticles produced by CS-TPP 7:1 ratio caused a decrease in the cell viability and affected cell morphology. This may be due to the strong electrostatic interaction between the cell membranes and positively charged nanoparticles^35^. Based on size, charge, absorption, and effect on cell viability, nanoparticles prepared using a 5:1 ration of CS-TPP and dsRNA were used in subsequent experiments.

### Characterization of CS-TPP-dsRNA nanoparticles

**Figure 1:**
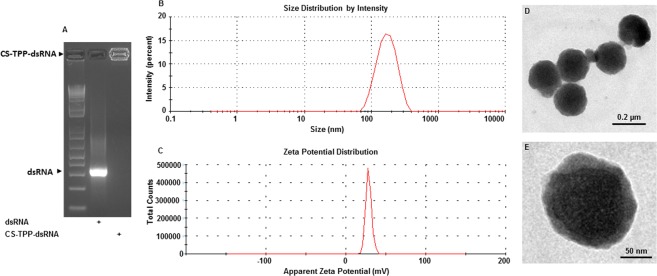
Preparation and characterization of CS-TPP-dsRNA nanoparticles. (**A**) The formation of CS-TPP-dsRNA complexes was verified by agarose gel electrophoresis. 1 kb plus ladder, naked dsRNA and CS-TPP-dsRNA complexes were resolved on 1% (w/v) agarose gel, stained with GelRed® and photographed under UV light. The picture of the gel shows differences in the migration of free dsRNA and CS-TPP-dsRNA complexes. (**B**,**C**) The mean particle diameter (z-average), polydispersity (PDI), and zeta potential (surface charge) of freshly prepared CS-TPP-dsRNA nanoparticles were determined by photon correlation spectroscopy (PCS) using Zetasizer (Malvern Instruments, UK). All measurements were performed in triplicate at 25 °C and data are represented as mean ± standard deviation. (**D**,**E**) Morphological characterization of CS-TPP-dsRNA nanoparticles was carried out by Transmission electron microscopy. A drop of CS-TPP-dsRNA nanoparticles on the copper microgrid was natively stained with 2% phosphotungstic acid and photographed under a TEM (HRTEM, JEOL 2010F, Japan).

Gel retardation assays of the CS-TPP-dsRNA nanoparticles prepared showed efficient incorporation of dsRNA into nanoparticles (Fig. 1A). The mean particle size of CS-TPP-dsRNA is less than 200 nm and a lower PDI of 0.207 as determined by DLS (Fig. 1B). The surface charge of CS-TPP-dsRNA nanoparticles is +34.37 ± 0.94 mV (Fig. 1C). The TEM images of CS-TPP-dsRNA nanoparticles showed a spherical structure (Fig. 1D,E). The possibility of nanoparticles modifying the surface morphology is of particular importance for *in vivo* applications. It has previously been reported that particles in the nanometer size and of the spherical structure have a relatively higher intracellular uptake compared to microparticles^36^. In our studies, approximately 80% entrapment efficiency was observed as measured by UV-visible spectrophotometry. Previous studies showed that the entrapment efficiency of siRNA loaded onto nanoparticles decreased significantly by increasing CS concentration. Inefficient siRNA entrapment was noted when higher concentrations of CS were used as the viscous solution restricted the association of the siRNA^37^. The low entrapment efficiency of nanoparticles may be due to interference shielding effects, which affect the interaction between nucleic acid and amino groups of CS^38^.

**Figure 2:**
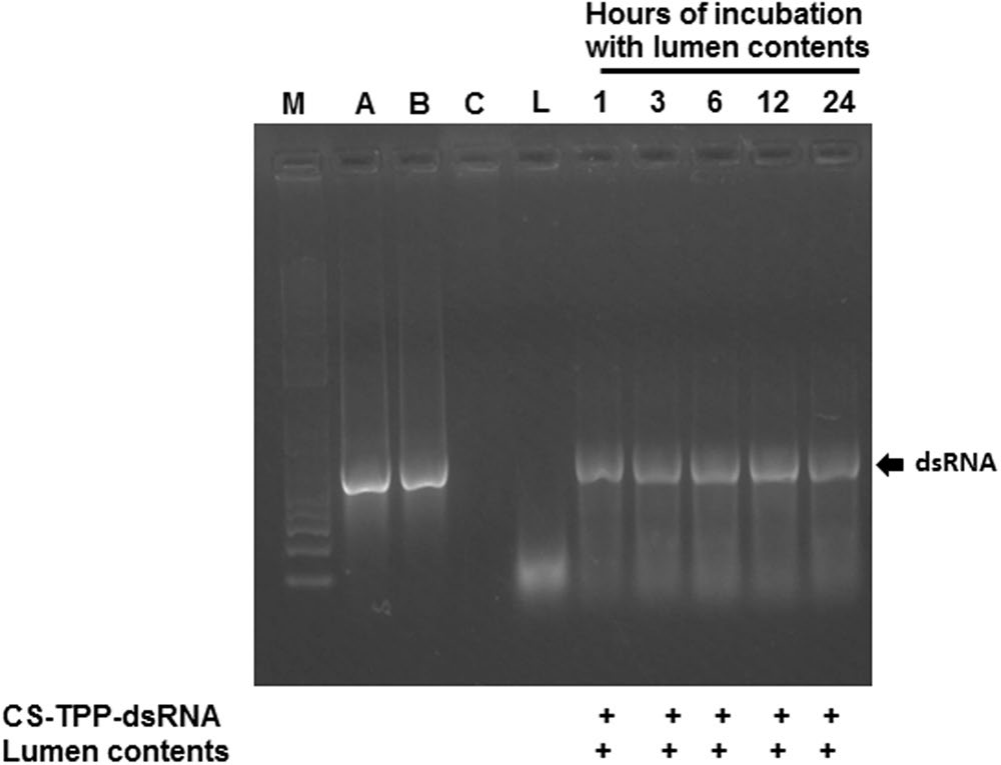
Stability of CS-TPP-dsRNA nanoparticle complexes exposed to lumen contents of mosquito larvae was assessed by gel electrophoresis. CS-TPP-dsRNA nanoparticles were exposed to lumen contents collected from *Aedes aegypti* larvae. At 1, 3, 6, 12 and 24 h after mixing dsRNA and lumen contents, the samples were collected and resolved on 1% agarose gels. The gels were stained with GelRed® and photographed under UV light. M, 1Kb plus DNA ladder; A, Naked dsRNA; B, dsRNA dissociated from CS-TPP-dsRNA; C, CS-TPP NP and L, lumen contents.

One of the most important factors governing RNAi efficiency is the capacity of a carrier system to protect dsRNA from nuclease degradation. To investigate the nuclease protection ability of CS-TPP-dsRNA nanoparticles, the nanoparticles prepared were exposed to the lumen contents of the alimentary canal dissected from mosquito larvae. The nucleases present in the lumen of mosquito larvae degraded naked dsRNA within one hour of exposure^39^. In contrast, the CS-TPP-dsRNA nanoparticles protected dsRNA from nuclease degradation up to 24 h (Fig. 2). In addition, dsRNA was dissociated from CS-TPP nanoparticles with the help of heparin (1000 U-ml). The dsRNA stability was analyzed by gel electrophoresis. As shown in Fig. 2, the dsRNA in CS-TPP-dsRNA complexes was protected from digestion by nucleases. The average band intensity in gels was determined and shown in Fig. S2. The intensity of bands was not significantly different confirming that the dsRNA in CS-TPP-dsRNA complexes was protected from digestion by nucleases.

CS-TPP-dsRNA nanoparticles were stored at various temperatures of 4 °C, 25 °C and 37 °C in deionized water up to 10 days and analyzed by gel electrophoresis. As shown in Fig. S3, no reduction in CS-TPP-dsRNA complexes were  detected. A previous study revealed that cross-linkers can enhance the stability of particulates^40^. We found that nanoparticle size increased after 10 days of storage. These results are similar to reports on CS-TPP-siRNA nanoparticles, which exhibited a slight increase in particle size after 15 days of storage^24^. The release profile of dsRNA from CS-TPP was studied in PBS at pH 7.4 up to 60 h. dsRNA was rapidly released during  the first 30 h, which resulted in a 39% cumulative release of dsRNA (Fig. S4). After 30 h, the dsRNA was slowly released up to 60 h, resulting in a 55% cumulative dsRNA release (Fig. S4). Cross-linking may be responsible for the strong interaction between CS-TPP-dsRNA nanoparticles.

### *In vivo* biodistribution and internalization of CS-TPP-dsRNA nanoparticles

**Figure 3:**
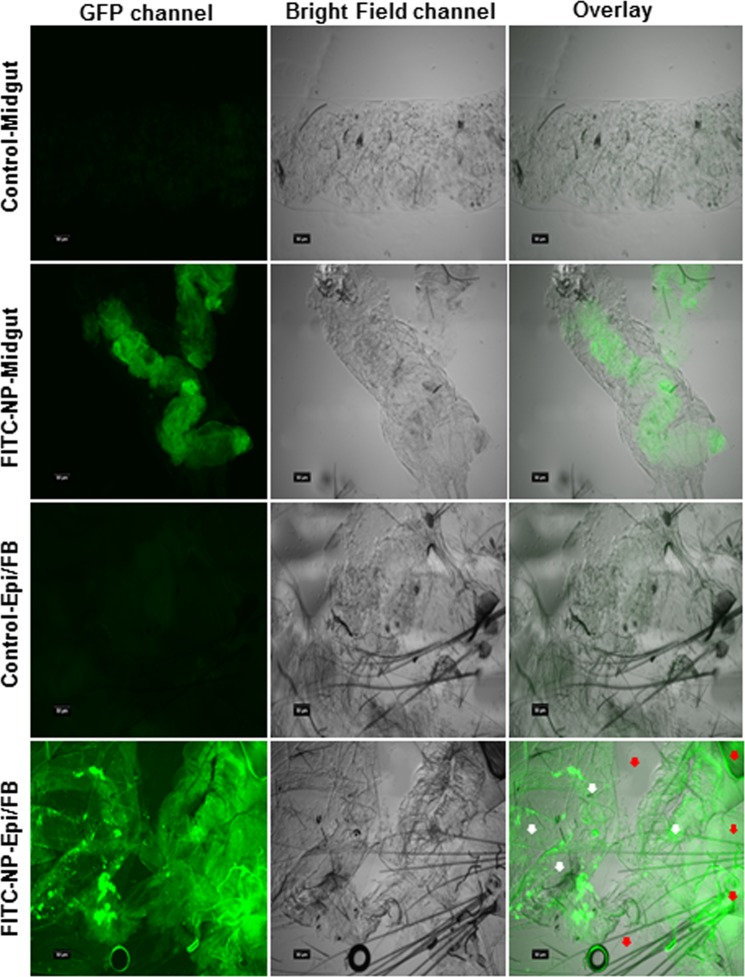
Fluorescence microscope analysis of the distribution of FITC labeled CS-TPP nanoparticles in mosquito larvae. CS-TTP-dsRNA nanoparticles labeled with FITC fluorescein dye were fed to mosquito larvae. At 24 h after feeding of nanoparticles, the midgut and epidermis with the attached fat body were dissected. The tissues were washed with 1X PBS buffer and then visualized under Nikon ECLIPSE 90i fluorescence microscopy. White and red arrows indicate fat body (FB) and epidermis (Epi) tissues, respectively. (Scale bar = 50 µm).

**Figure 4:**
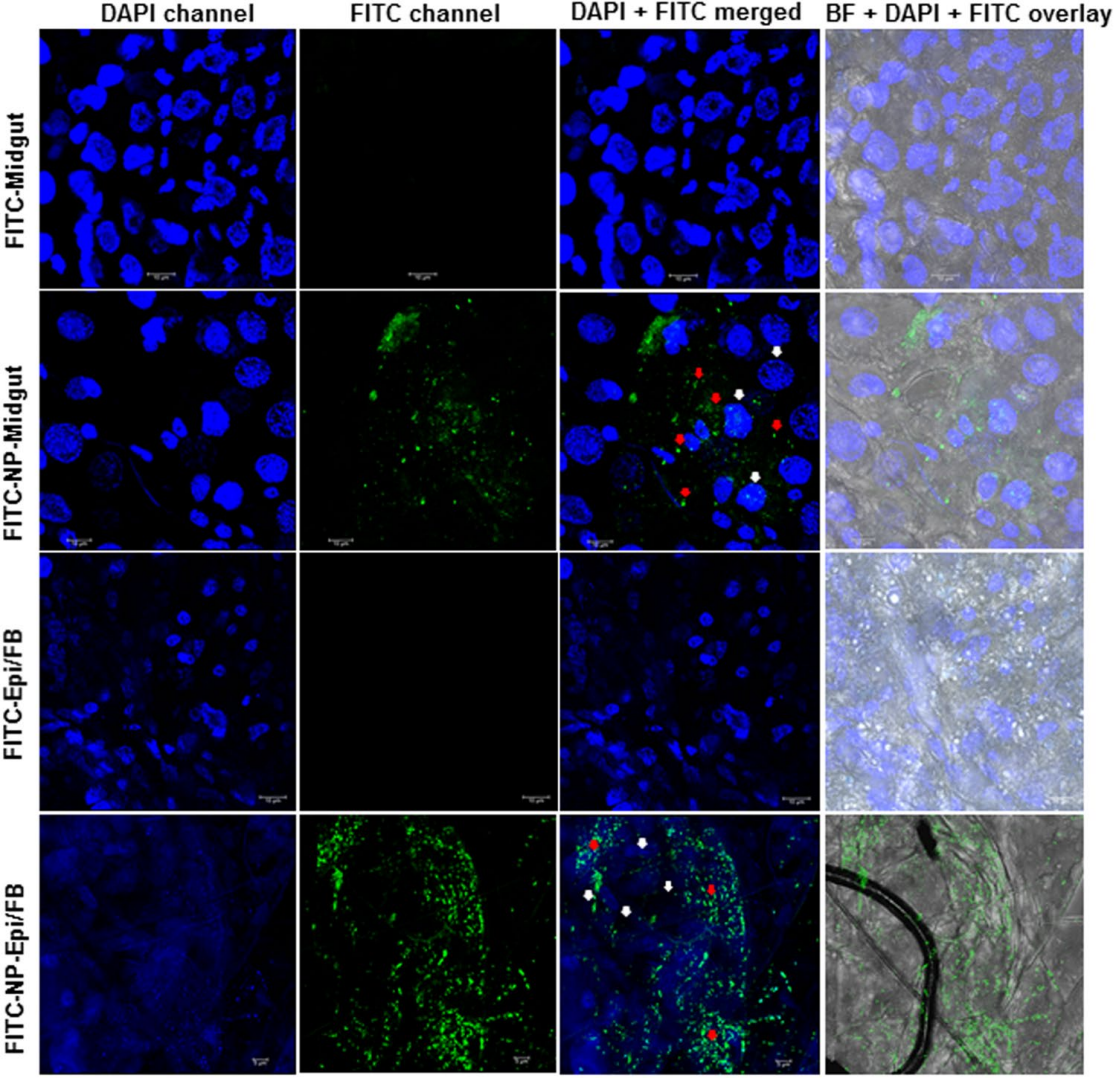
Confocal microscope analysis of biodistribution of FITC labeled CS-TPP nanoparticles in mosquito larvae. CS-TPP-dsRNA nanoparticles conjugated with FITC fluorescein dye were fed to mosquito larvae. At 24 h after feeding of nanoparticles, midgut and epidermis with the attached fat body were dissected. The tissues were washed with 1X PBS buffer and fixed with 4% paraformaldehyde. Tissues were stained with mounting medium containing DAPI and visualized under 63X magnification of Leica SP8 confocal microscope. The white and red arrow indicates nucleus and nanoparticles, respectively (Scale bar = 20 µm, Top three panels) and 5 µm, **Bottom panel**).

An overall positive charge of the nanoparticle complex formed under optimal conditions fosters cell attachment, followed by membrane fusion via endocytosis, and ultimately endosomal escape by proton sponge effect^41^. We studied *in vivo* biodistribution and internalization of nanoparticles in mosquito larvae using FITC-labeled CS-TPP-dsRNA nanoparticles. At 24 h post feeding, whole larvae examined under a fluorescence microscope showed clear green signals of FITC-labeled nanoparticles in the whole larval body. The clear fluorescence signals were visualized in both fat body attached to the epidermis and midgut tissues, whereas no fluorescence signals were observed in the control tissues (Fig. 3). Confocal microscopy analysis of the tissues dissected from FITC-labeled nanoparticles revealed fluorescence signals representing FITC-labeled nanoparticles internalized in the midgut and fat body cells (Fig. 4). Conversely, the tissues dissected from FITC-fluorescein fed larvae did not show any such signals (Fig. 4). Previous studies showed that both positively and negatively charged nanoparticles were distributed throughout the larval body, but positively charged nanoparticles exhibited faster attachment or internalization in tissues. Indeed, positively charged nanoparticles were present in the gastrointestinal tract within the gastric caeca. Although negatively charged nanoparticles were detected even after adult metamorphosis in tissues associated with the head, body parts, and ovaries, *in vitro* studies found that positively charged nanoparticles were more effective than negatively charged nanoparticles in mosquito larvae^42,43^. Our data showing the distribution of positively charged nanoparticles in the midgut, fat body and epidermis are in line with these published reports. In mammalian cells, positively charged nanoparticles are internalized more efficiently than negatively charged nanoparticles due to their electrostatic interactions with the plasma membrane^44^. Indeed, positively charged CS-TPP nanoparticles were shown to deliver nucleic acids into HeLa cells^36^.

### The efficacy of CS-TPP-dsRNA nanoparticles

**Figure 5:**
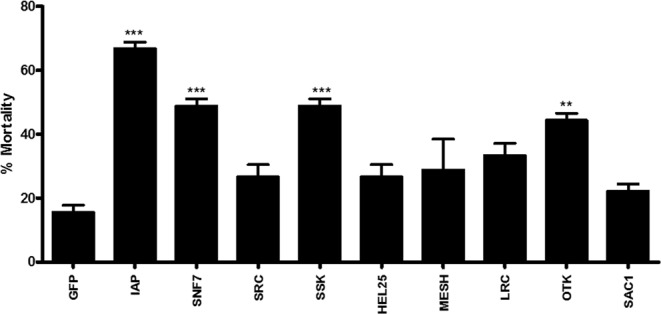
Screening candidate genes to control mosquito larvae by delivering dsRNA conjugated to CS-TPP nanoparticles. Mosquito larvae were fed on CS-TPP-dsRNA nanoparticles containing dsRNA targeting nine selected genes or GFP used as a control. Mortality was recorded on the 10th day after initiation of feeding. Mean ± S.E (n = 3). The asterisks above the bar indicate the significance of difference (One-way ANOVA, Turkey’s test **P< 0.05, ***P< 0.001). GFP, green fluorescence protein; IAP, an inhibitor of apoptosis; SNF7, vacuolar-sorting protein; SRC, steroid receptor co-activator; SSK, Snakeskin; HEL25E, Helicase at 25E; MESH, membrane-spanning protein; LRC, leukocyte receptor complex member; OTK, offtrack and SAC1, suppressor of actin.

**Figure 6:**
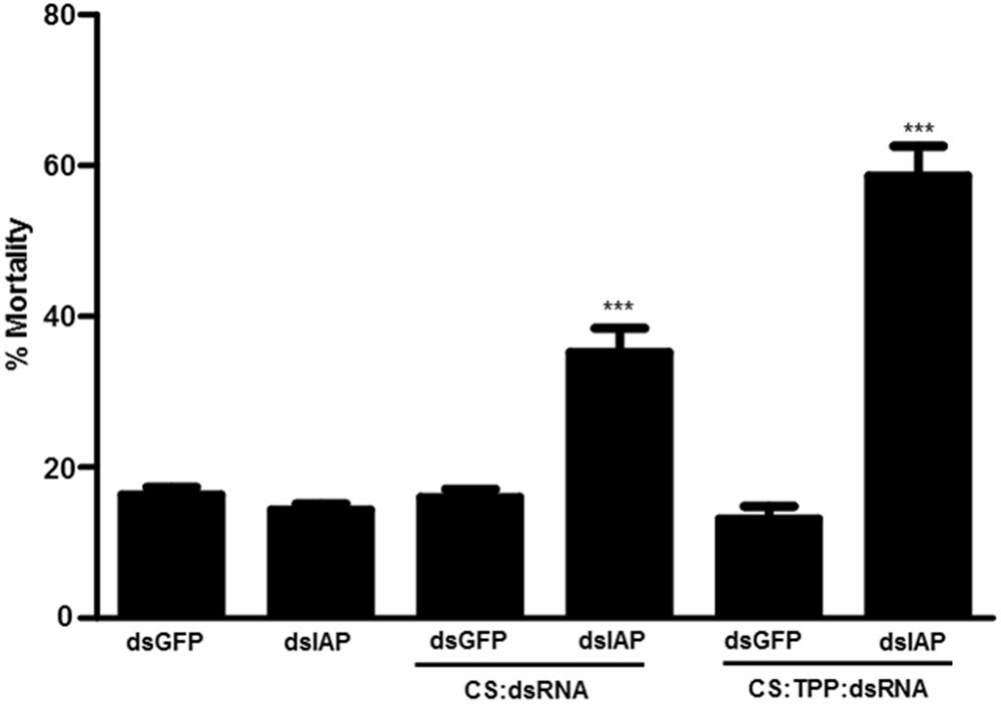
dsIAP conjugated to CS and CS-TPP nanoparticles induce mortality in mosquito larvae. Mosquito larvae were fed on diet containing naked dsIAP, dsGFP, CS-dsIAP, CS-dsGFP, CS-TPP-dsIAP or CS-TPP-dsGFP nanoparticles. Mortality was recorded on the 10th day after initiation of feeding. Mean ± S.E (n = 3). The asterisks above the bar indicate the significance of difference (One-way ANOVA, Turkey’s test ***P< 0.001).

**Figure 7:**
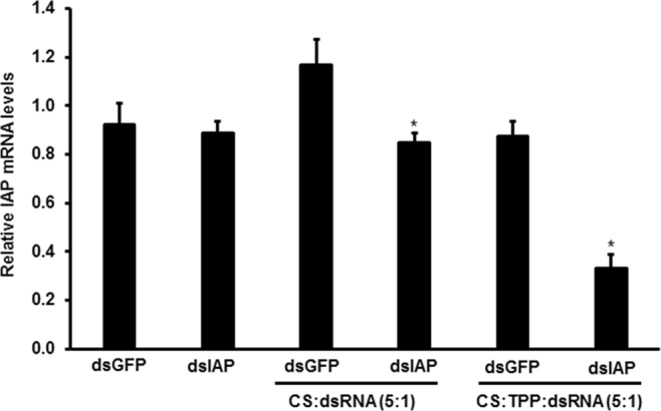
CS-TPP-AaIAP dsRNA nanoparticles trigger efficient knockdown of the AaIAP gene. Knockdown of AaIAP gene was analyzed in mosquito larvae fed with naked dsIAP, CS-TPP-dsIAP or CS-TPP-dsGFP nanoparticles by qRT-PCR. Five days after feeding naked dsIAP, dsGFP, CS-dsIAP, CS-dsGFP CS-TPP-dsIAP or CS-TPP-dsGFP nanoparticles to *Aedes aegypti* larvae; the RNA was isolated, converted to cDNA and used in qRT-PCR to determine relative IAP mRNA levels. Data are presented as mean ± SE. (n = 3). The asterisks above the bar indicate the significance of difference (T-TEST, ***P< 0.001).

The effectiveness of CS-TPP-dsRNA nanoparticles in silencing target genes and killing mosquito larvae was tested. Nine candidate genes (Table S4) were selected based on their effectiveness in triggering RNAi in *Aedes aegypti* and other insects tested. The dsRNA prepared using fragments of nine selected genes and the gene coding for enhanced green fluorescence protein (EGFP) as a control were used to prepare CS-TPP-dsRNA nanoparticles. The nanoparticles, mosquito larval food, and agarose were used to prepare food pellets. The food pellets were fed to larvae once a day until pupation. The mortality caused by CS-TPP-dsRNA nanoparticles varied from 20–65% depending on the gene targeted. The variation in mortality caused by CS-TPP-dsRNA nanoparticles targeting different genes may be related to the function of these target genes. The control larvae fed on CS-TPP-dsGFP showed 18% mortality. The CS-TPP-dsRNA nanoparticles targeting IAP, SNF7, SSK, and OTK caused significantly higher mortality when compared to the mortality caused by control CS-TPP-dsGFP nanoparticles (Fig. 5). We then compared naked dsIAP, CS-dsIAP and CS-TPP-dsIAP for their ability to induce mortality in *Ae. aegypti* larvae. Compared to >60% mortality induced by CS-TPP-dsIAP nanoparticles, the CS-dsIAP nanoparticles induced only 35% mortality and the naked dsIAP did not induce significant mortality (Fig. 6). We determined the IAP gene knockdown efficiency using reverse-transcriptase quantitative real-time PCR (RT-qPCR). The mRNA levels of IAP target gene were quantified on the fifth day after initiation of feeding naked dsIAP, dsGFP, CS-dsIAP, CS-dsGFP, CS-TPP-dsGFP or CS-TPP-dsIAP. Oral administration of CS-TPP-dsIAP or CS-dsIAP nanoparticles reduced the IAP mRNA levels by 62% and 27% respectively when compared to the levels in control larvae treated with CS-TPP-dsGFP or CS-dsGFP (Fig. 7). The naked dsIAP feeding did not cause knockdown on iap gene. These results suggest that feeding mosquito larvae with CS-TPP-dsRNA nanoparticles can deliver dsRNA to their cells, resulting in uptake of dsRNA and suppression of target gene expression. The iap gene from *Bombyx mori* was identified and shown to function as a caspase inhibitor to block apoptosis^45^. The iap1 gene was identified in *Ae. aegypti* and showed that the gene product inhibits both initiator and effector caspases^46^. In Aag-2 cells, five genes coding for IAPs (1, 2, 5, 6 and 9) were identified. Treating these cells with dsRNA targeting these genes caused a significant reduction in target gene mRNA levels, but only dsIAP1 induced apoptosis phenotype^47^. Knockdown of iap gene caused mortality in *Lygus lineolaris Halyomorpha halys, Agrilus planipennis*, and *Anoplophora glabripennis*^48–51^.

## Conclusions

The previous studies^20–22,52^ used CS nanoparticles to deliver dsRNA to silence genes in insects, including *Ae. aegypti*. However, the knockdown efficiency and mortality achieved by CS-mediated delivery of dsRNA were not very high; therefore, these nanoparticles are not widely used to deliver dsRNA to mosquitoes and other insects. In our previous studies in *Ae. aegypti* larvae, CS nanoparticle-based delivery of dsRNA caused less than 50% knockdown of the target gene and larval mortality^52^. In this study, we found that CS-TPP-dsRNA nanoparticles are better than CS nanoparticles in delivering dsRNA to mosquito larvae. In future studies, it may be possible to achieve up to 100% mortality of larvae after modifying strong bond interaction of nanoparticles and identifying an effective target gene. Work is in progress to screen and identify effective target genes and to determine the best delivery system to achieve 100% mortality of *Ae. aegypti* larvae after delivery of dsRNA using nanoparticles.

## Materials and Methods

### Visual inspection of CS-TPP nanoparticles formation

The formation of CS-TPP nanoparticles was examined by visual inspection^27,28^. A preliminary test was done to determine the optimum conditions required for the formation of nanoparticles. In this regard, medium molecular weight CS (75–85% deacetylated) was dissolved in 1% acetic acid (w/v) to prepare 10 mg/ml solution. The TPP was dissolved in deionized water (0.25–1.5 mg/ml). Visualization of the nanoparticle formation was carried out by the addition of 2 ml of TPP solution to 5 ml of CS solution under magnetic stirring at room temperature.

### Nanoparticles preparation

CS-TPP-dsRNA nanoparticles were formed using the ionic gelation method. Nanoparticles were prepared by adding 1 ml of TPP aqueous solution (1 mg/ml) and 1 ml of dsRNA (0.250 mg/ml) in deionized water in a dropwise manner to 5 ml of CS solution (1 mg/ml) under magnetic mixing at room temperature. The nanoparticles were then incubated for 30 min at room temperature. Nanoparticles were collected by centrifugation at 13,000 × g for 10 min. The supernatant was removed, and the pellet was washed three times with deionized water and resuspended in MilliQ ultrapure water. The nanoparticles were then sonicated for 5 min in an ultrasonic liquid processor and used for further analysis.

### Gel retardation assay

The binding affinity of dsRNA to CS-TPP nanoparticles was confirmed by agarose gel electrophoresis using a 1 kb plus DNA ladder for size reference. Naked dsRNA and CS-TPP nanoparticles were used as positive and negative controls, respectively. The CS-TPP-dsRNA nanoparticles were loaded onto 1% agarose gels stained with GelRed® (Biotium, USA). After electrophoresis, the gel was photographed with an Alpha Imager™ Gel Imaging System (Alpha Innotech, San Leandro, CA) under ultraviolet light.

### Dynamic light scattering (DLS) analysis

The mean particle size (Z-average), surface charge (Zeta potential) and polydispersity (PDI) of CS-TPP-dsRNA nanoparticles were measured by photon correlation spectroscopy (PCS) using Zetasizer (Malvern Instruments, UK). All measurements were made in triplicate at 25 °C and data are reported as a mean ± standard deviation.

### TEM imaging of CS-TPP-dsRNA nanoparticles

To determine the morphology of the nanoparticles, the particles were viewed under a transmission electron microscope (TEM). A drop of CS-TPP-dsRNA nanoparticles was fixed on a copper microgrid that was natively stained with 2% phosphotungstic acid. The stained nanoparticles were incubated for 10 min at room temperature. The nanoparticles were then viewed under a TEM (HRTEM, JEOL 2010F, Japan) and images were captured.

### Determination of dsRNA loading efficiency

To determine the dsRNA loading efficacy of nanoparticles, the dsRNA conjugated with nanoparticles was separated from free dsRNA by centrifugation. The free dsRNA in the supernatants was measured by its absorbance at 260 nm wavelength using NanoDrop-2000 spectrophotometer (Thermo Fisher Scientific Inc., Waltham, MA). The amount of dsRNA incorporated within the nanoparticles was calculated by the difference between the initial quantity of dsRNA (Total dsRNA) and the remaining amount in the supernatant (Free dsRNA). The supernatant recovered from naked nanoparticles was used as a blank. Entrapment efficiency was calculated using the following method^53^: Entrapment efficiency = Total dsRNA – Free dsRNA/Total dsRNA x 100.

### dsRNA stability assay

To investigate *ex vivo* degradation, an assay described recently was used^54^. Mosquito larvae entire midgut was dissected and placed in 100 µl of 1X PBS and centrifuged for 10 min at 20,000 × g. The supernatant was then collected and centrifuged in the same manner. Ten microliters of nanoparticles containing 1 µg of dsRNA were added to 10 µl (1 µg) of midgut extract and samples were collected at various time points (1, 3, 6, 12 and 24 h). Naked dsRNA was incubated in the midgut extract for 1 h as a control. The samples were resolved on a 1.0% (w/v) agarose gel, stained with GelRed® (Biotium, USA) and photographed with an Alpha Imager™ Gel Imaging System (Alpha Innotech, San Leandro, CA) under ultraviolet light.

### Storage stability of CS-TPP-dsRNA nanoparticle complexes

CS-TPP-dsRNA nanoparticles were suspended in nuclease-free water and stored at 4 °C, 25 °C and 37 °C for 10 days. Nanoparticle stability was determined by 1% agarose gel electrophoresis and nanoparticle size was measured at previously established time points.

### *In vitro* release studies

The CS-TPP-dsRNA nanoparticles were incubated at room temperature (25 °C), in 2 ml of 1x PBS at pH 7.4. At certain intervals, (10, 20, 30, 40, 50, and 60 h) nanoparticles were centrifuged and 1.5 ml of the supernatant removed and replaced by 1x PBS solution. The amount of dsRNA released from the nanoparticles was determined using the NanoDrop-2000 spectrophotometer (Thermo Fisher Scientific Inc., Waltham, MA).

### Biodistribution of FITC labeled nanoparticles

*In vivo*, biodistribution of nanoparticles was investigated using FITC-labeled nanoparticles. The FITC-labeled nanoparticles were prepared by following the method described previously^55^. Five mosquito larvae were placed in each well of a 24-well plate containing 1 ml of nuclease-free water. FITC-nanoparticles were evenly mixed with mosquito larval food and embedded in 1% melted agarose. This food pellet was fed to mosquito larvae under dark conditions. After 24 h, the whole larvae were viewed under 4X magnification of fluorescence microscope (Nikon ECLIPSE 90i). For biodistribution studies, mosquito larvae were dissected, and the alimentary canal and fat bodies attached to the epidermis were collected and washed with 1X PBS. The tissues were visualized under 10X magnification of a fluorescence microscope (Nikon ECLIPSE 90i).

### Cellular Internalization of nanoparticles by confocal microscopy

FITC labeled nanoparticles were fed to the mosquito larvae, and at 24 h post feeding, the midgut and epidermis attached with fat body tissues were dissected and washed with 1X PBS buffer. The tissues were fixed with 4% paraformaldehyde solution and incubated at 4 °C overnight under dark conditions. The fixed tissues were mounted on microscope slides stained with EverBrite™ mounting medium containing DAPI (Biotium, Inc. Fremont, CA) and examined under 63X magnification in a confocal laser-scanning microscope (Leica TCS SP8) using DAPI, FITC (490–525 wavelength) and bright field (BF) channels.

### Mosquito rearing

*Aedes aegypti* (Waco strain) mosquitoes were reared as described previously^27^. Eggs were collected from lab colony adults and stored dry for approximately 2–4 weeks before hatching. Eggs were hatched in a 64 oz plastic pan containing 300 mL deoxygenated, filtered water inoculated with 10 mL of bovine live powder feeding solution (60 g/L). The pans were maintained in an incubator at 27 ± 1.0 °C under a photoperiodic regime of 16:8 hour (L: D). Freshly molted second-instar larvae were collected and briefly held in a separate pan containing filtered water before being transferred to 24-well plates for bioassays.

### dsRNA synthesis

Nine candidate genes were selected based on previous studies of their efficacy as RNAi triggers^47,52,56,57^. The dsRNA targeting these genes was *in vitro* synthesized using the MEGAscript  T7 Transcription Kit (Ambion Inc., Foster City, CA USA) as described previously^55^. Briefly, 300–500 bp fragment of each gene was PCR amplified using gene-specific primers (Table S4) containing T7 RNA polymerase sequence at the 5′ end. 500 ng of the purified PCR product was used as a template in 20 μL *in vitro* transcription reaction. The reactdsRNAion mix was incubated for 16 h at 37 °C, followed by 30 min of DNase I treatment. The reaction mixture was heat inactivated at 70 °C for 10 min and cooled down slowly to room temperature. The dsRNA was precipitated by adding 0.1x volume of sodium acetate (3 M, pH 5.2) and 2.5x volumes of 100% ethanol and then kept at −20 °C for at least 2 h. The reaction contents were then centrifuged at 4 °C for 15 min. The dsRNA pellet was rinsed with 75% ethanol and centrifuged again at 4 °C for 5 min. The ethanol was removed and the dsRNA pellet was dried and resuspended in MilliQ ultrapure water. The quality and quantity of dsRNA were checked by agarose gel electrophoresis and NanoDrop-2000 spectrophotometer (Thermo Fisher Scientific Inc., Waltham, MA), respectively.

### Mosquito feeding assay

Mosquito larval food containing nanoparticles was prepared by following methods described previously^20^. Briefly, 50 µl of nanoparticles containing 40 µg of dsRNA were mixed with 5 mg of bovine liver powder and added to 1.5% pre-melted agarose gel solution at 55 °C. A group of 5–7-second instar larvae were transferred to each well of 24-well plate containing 1 ml of deionized water. Each treatment was replicated three times, and each experiment was repeated at least five times. The food pellet containing 40 µg of dsRNA was divided into three equal pieces and distributed to each well. Mortality was recorded until mosquito larvae in the control group became adults. The transcript levels of the dsIAP target gene were determined on the 5th day after the first feeding of dsRNA.

### Quantitative Real-Time PCR (RT-qPCR)

Total RNA was isolated from mosquito larvae using TRIzol reagent (Molecular Research Center Inc., Cincinnati, OH) following the manufacturer’s protocol. The total RNA was then treated with DNase I (Ambion Inc., Austin, TX). Two µg of total RNA was used for first strand cDNA synthesized using M-MLV Reverse Transcriptase (Invitrogen, USA). The first strand cDNA was used as a template for qPCR analysis. Each 10 µl qRT-PCR (Applied Biosystems, USA) reaction contained 5 µl of Fast Start SYBR Green Master mix (Roche Diagnostics, Indianapolis), 2 μl of 1:2 diluted cDNA and 0.2 μl each of 10 µM forward and reversed gene-specific primers (Table S4). An initial incubation of 95^o^ C for 3 min, followed by 40 cycles of 95 for 10 sec, 55 for 20 sec and 72 for 30 sec settings, were used. Each experiment was replicated a minimum of three times using the samples from independent treatments. Relative expression levels of a target gene were determined using the reference gene, S7RP the 2^−ΔΔCT^ method^58^.

### Statistical analysis

The nanoparticle characterization data (size, charge, and PDI) are shown as the Mean ± STD. The statistical significance of gene expression analysis for qRT-PCR was determined using a t-test analysis. A one-way analysis of variance (ANOVA) was used to determined larval mosquito mortality, and their significance was compared by Turkey’s multiple comparison test. P values of <0.05 were considered significant. The analyses were performed using GraphPad Prism 5 (La Jolla, CA) for windows.

## Supplementary information

Suppl.Info
