# Supplementary material for: Development of CS-TPP-dsRNA nanoparticles to enhance RNAi efficiency in the yellow fever mosquito, Aedes aegypti
Source: Sci Rep. 2019 Jun 19;9:8775. doi: 10.1038/s41598-019-45019-z (PMC6584730; doi:10.1038/s41598-019-45019-z)
Supplement: Supplementary file 1 — Suppl.Info [file 41598_2019_45019_MOESM1_ESM.pdf]

Supplementary Information

Development of CS-TPP-dsRNA nanoparticles to enhance RNAi efficiency in the yellow fever mosquito, *Aedes aegypti*

Ramesh Kumar Dhandapani, Dhandapani Gurusamy, Jeffrey L. Howell and Subba Reddy Palli*

Table S1 Formation of the chitosan and TPP nanoparticles

	TPP (mg/ml)					
CS (mg/ml)	0.25	0.5	0.75	1	1.25	1.5
1 mg	++	++	++	++	--	--
3 mg	××	××	++	++	++	++
5 mg	××	××	++	++	++	++
7 mg	××	××	××	++	++	++

++, opalescent suspension; ××, Clear solution; --, aggregates

Table S2 DLS analysis of CS-TPP nanoparticles

**Ratio**	**Size**	**Charge**	**PDI**
CS-TPP (1:1)	160±3.21	1.97±0.29	0.45±0.018
CS-TPP (3:1)	171±2.31	13.97±1.51	0.29±0.021
CS-TPP (5:1)	115±3.71	48.67±1.71	0.19±0.023
CS-TPP (7:1)	186.67±2.96	65.73±1.68	0.51±0.015

Table S3 DLS analysis of CS-TPP-dsRNA nanoparticles

**Ratio**	**Size (nm)**	**Charge (mv)**	**PDI**	**Percent dsRNA incorporated**
CS-TPP-dsRNA (1:1)	242.67±4.06	-18.37±1.97	0.31±0.04	32±2.31
CS-TPP-dsRNA (3:1)	263.33±5.61	11±0.93	0.39±0.02	61.67±2.03
CS-TPP-dsRNA (5:1)	181.67±3.48	34.37±0.94	0.26±0.02	81.33±2.03
CS-TPP-dsRNA (7:1)	3157±9.29	47.57±1.25	0.46±0.03	68±1.73

Table S4. List of primers used in this study. All primers except those labeled as qPCR were used for dsRNA synthesis.

S. No	Genes	Primer sequences (5'-3')	Amplicon Size (bp)	Accession No	References
1.	EGFP	FP: CGATGCCACCTACGGCAA<br>RP: TGTCGCCCTCGAACTTCA	248		
2.	IAP1	FP: CTTCTGCCGAGTGGAAATCGG<br>RP: ATATTCCGGTAGCTTCTGTTG	349	DQ993355.1	47
		qPCR-FP: GTGTTTGGCCAAGAAGGAAAG<br>qPCR-RP: TGACTGAAGCGAGGATGTTG	118		
3.	SNF7	FP: ACGATGTCCACGAGATGATG<br>RP: CAGGCAGATCGGTTGCT	222	XM_001659907.2	52
4.	SRC	FP: CGTCAAATGCAGCAGATCACCCAA<br>RP: TGTTGGTTGTTCGAGGGAGAAGGT	431	XM_021845577.1	
5.	SSK	FP: TCATCTACCGAACGGGCTA<br>RP: TTCTCTCCGAGGTGATGTG	303	XM_021848746.1	56
6.	MESH	FP: TGGAACGGTAGTCAACATCA<br>RP: CCCACAGCGAGATCTGAAC	350	XM_021840549.1	
7.	HEL25E	FP: CAAGCTGTGCTAGGAATGGA<br>RP: CGAGAATGAAATGCTTCAGGTG	355	XM_001658256.2	
8.	SAC1	FP: ATACGGTCGACAGGTTCTGG<br>RP: CAGACGTTCTTGAACAGCCA	420	XM_001658506.2	57
9.	LRC	FP: GCCAGACTTGAGCAAACCTC<br>RP: TTGTTGTTTGGCGTTAGCAG	327	XM_001658387.2	
10.	OTK	FP: GGTGCTGACCGAGGTACATT<br>RP: AGGACTTTTCCGGTAGGCAT	338	XM_021853098.1	
11.	S7RP	qPCR-FP: ACCGCCGTCTACGATGCCA<br>qPCR-RP: ATGGTGGTCTGCTGGTTCTT	131	CR938234.1	58

Figures

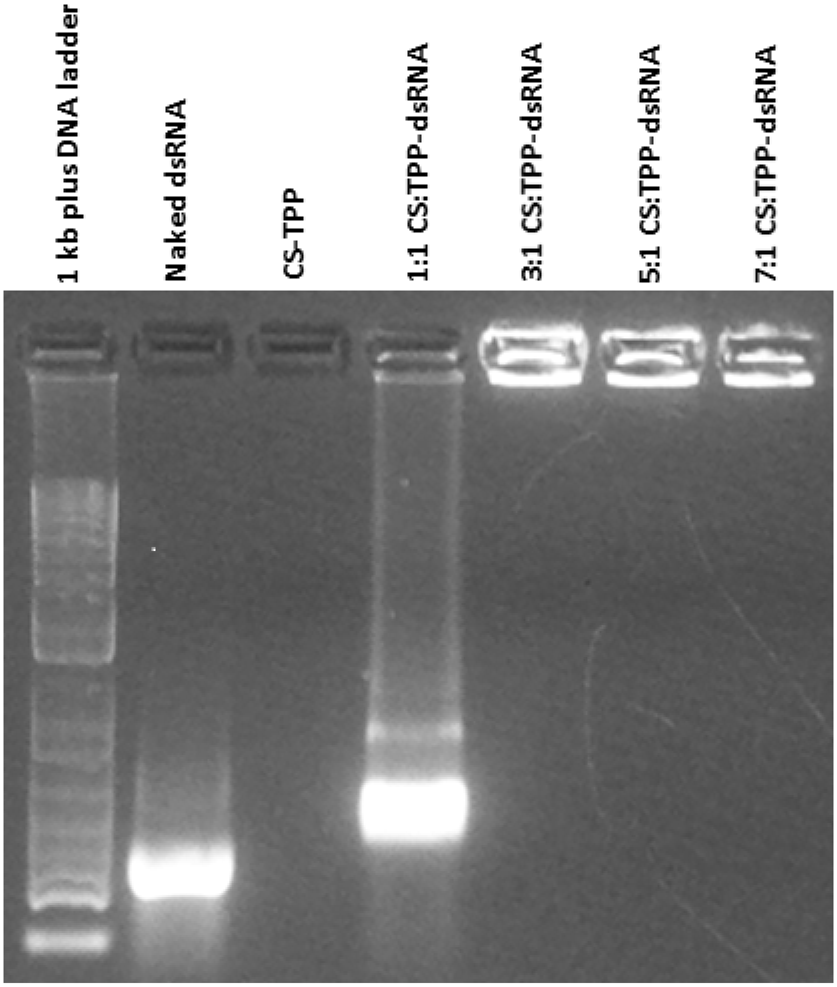

Fig S1 Gel retardation assay of nanoparticle complexes formed with different ratios of chitosan (CS). TPP and dsRNA were conjugated with different ratios of chitosan and analyzed by gel electrophoresis.

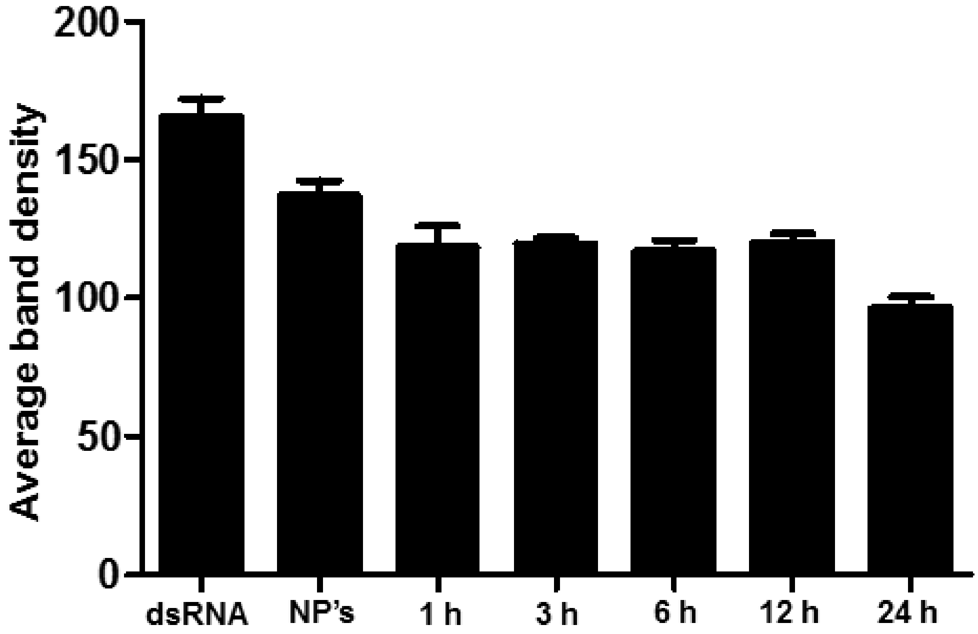

Fig S2 Stability of CS-TPP-dsRNA nanoparticle complexes exposed to lumen contents of mosquito larvae. CS-TPP-dsRNA nanoparticles were exposed to lumen contents collected from *Aedes aegypti* larvae. At 1, 3, 6, 12 and 24 h after mixing dsRNA and lumen contents, the samples were collected and analyzed in agarose gels. The gels were stained with GelRed® and photographed under UV light. The bands from scanned gel images were quantified and Mean+SE (n=3) are shown.

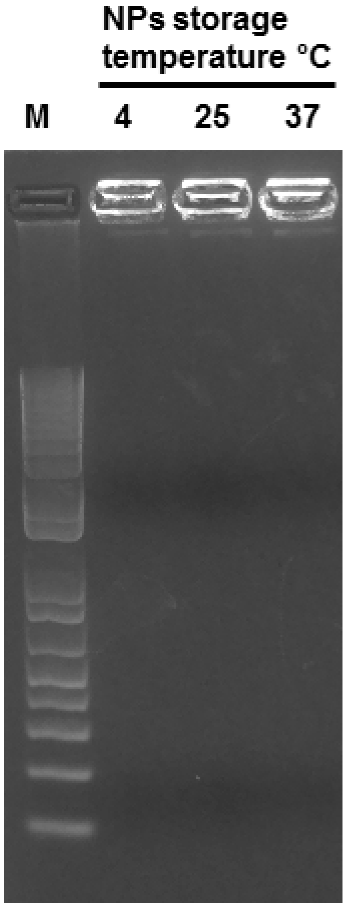

Fig S3 Storage stability of CS-TPP-dsRNA nanoparticles. NPs were prepared and stored at 4, 25 and 37°C for 10 days and analyzed by gel electrophoresis. M, 1 kb plus DNA ladder.

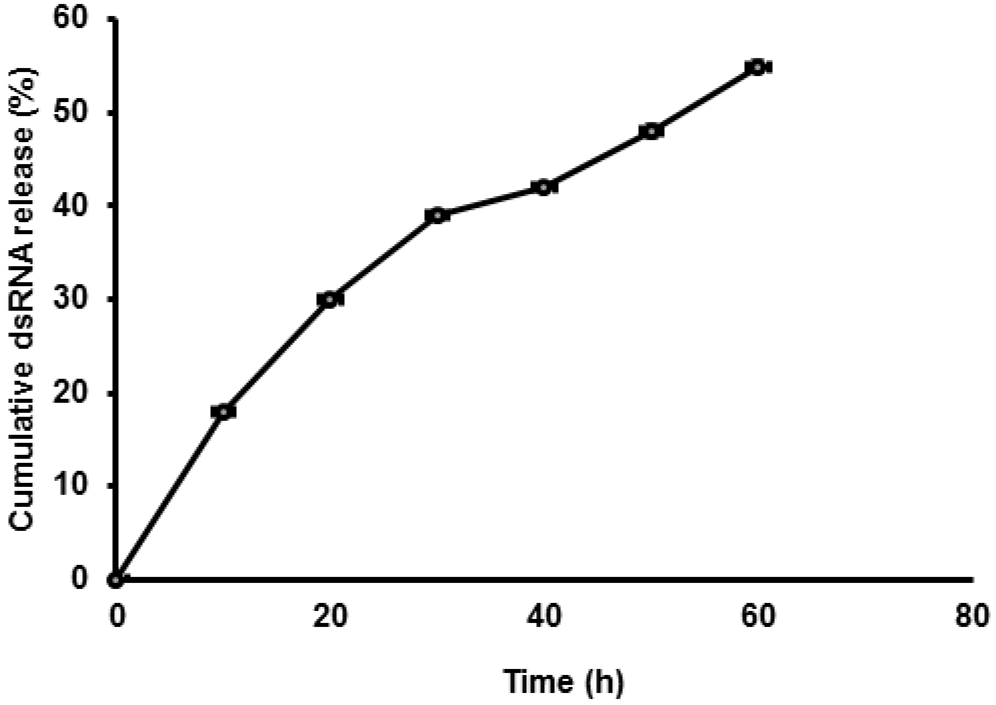

Fig S4 The profile of cumulative dsRNA release. CS-TPP-dsRNA NPs were incubated in 1X PBS buffer, pH 7.4, for different time intervals. The dsRNA released was quantified. The data shown are Mean ± SE (n=3).
